# Skin in the Game: An Assay to Monitor Leukocyte Infiltration in Dermal Lesions of a Guinea Pig Model for Tick-Borne Rickettsiosis

**DOI:** 10.3390/pathogens11020119

**Published:** 2022-01-20

**Authors:** Claire E. Cross, John V. Stokes, Navatha Alugubelly, Anne-Marie L. Ross, Bridget V. Willeford, Jamie D. Walker, Andrea S. Varela-Stokes

**Affiliations:** 1 Comparative Biomedical Sciences, College of Veterinary Medicine, Mississippi State University, Mississippi State, MS 39762, USA; clacross2@gmail.com (C.E.C.); jstokes@cvm.msstate.edu (J.V.S.); na413@msstate.edu (N.A.); ar2324@msstate.edu (A.-M.L.R.); 2 Laboratory Animal Resources, Mississippi State University, Mississippi State, MS 39762, USA; bmv2@msstate.edu (B.V.W.); jda16@msstate.edu (J.D.W.)

**Keywords:** *Cavia porcellus*, tick bite, tissue dissociation, innate immunity, strain 2, skin biopsy, flow cytometry

## Abstract

Intact, the skin typically serves as an effective barrier to the external world; however, once pathogens have breached this barrier via a wound, such as a tick bite, the surrounding tissues must recruit immune cells from the blood to neutralize the pathogen. With innate and adaptive immune systems being similar between the guinea pig and human systems, the ability of guinea pigs to show clinical signs of many infectious diseases, and the large size of guinea pigs relative to a murine model, the guinea pig is a valuable model for studying tick-borne and other pathogens that invade the skin. Here, we report a novel assay for assessing guinea pig leukocyte infiltration in the skin. Briefly, we developed an optimized six-color/eight-parameter polychromatic flow cytometric panel that combines enzymatic and mechanical dissociation of skin tissue with fluorescent antibody staining to allow for the immunophenotyping of guinea pig leukocytes that have migrated into the skin, resulting in inflammation. We designed this assay using a guinea pig model for tick-borne rickettsiosis to further investigate host–pathogen interactions in the skin, with preliminary data demonstrating immunophenotyping at skin lesions from infected ticks. We anticipate that future applications will include hypothesis testing to define the primary immune cell infiltrates responding to exposure to virulent, avirulent tick-borne rickettsiae, and tick-borne rickettsiae of unknown virulence. Other relevant applications include skin lesions resulting from other vector-borne pathogens, *Staphylococcus aureus* infection, and Buruli ulcer caused by *Mycobacterium ulcerans*.

## 1. Introduction

Skin is the largest organ of the human body, forming a robust, impermeable barrier to the outside world, with mechanical, chemical, and microbiological mechanisms to make it the first line of defense against invading pathogens [1]. A surface wound, skin lesion, or penetrating insult such as a tick bite, is typically required for a pathogen to breach the skin barrier. Once inside the body, the pathogen activates tissue-resident effector cells that release cytokines, stimulating an immune response that results in inflammation [2]. Secreted chemokines recruit various immune cells from the blood to the tissue in a process known as leukocyte infiltration. Inflammation of the skin can occur in response to various pathogens, including viral, bacterial, fungal, and protozoal or helminth agents. The leading cause of skin infections is *Staphylococcus aureus*, an agent of cutaneous abscesses and cellulitis [3]. Its importance has increased with the emergence of methicillin-resistant *S. aureus* (MRSA) strains. Another skin infection is the Buruli ulcer, caused by *Mycobacterium ulcerans*; there is a clear and ongoing need to understand better the mechanisms of the early immune response to *M. ulcerans* infection [4]. Importantly, vector-borne diseases require arthropods to transmit pathogens to hosts, which often occurs during arthropod feeding when mouthparts are inserted into host skin.

Tick-borne diseases, such as Lyme disease, spotted fever rickettsioses, and ehrlichiosis, make up over 75% of all vector-borne diseases reported in the United States; the number of reported cases of tick-borne disease has more than doubled since 2004 [5]. The host–tick–pathogen interactions that occur in the skin are not fully understood, and there is a need for assays to characterize the skin immune response to a tick bite [6]. Further, a suitable animal model is required to study host–tick–pathogen interactions effectively. The diseases already mentioned, including spotted fever rickettsiosis, and infectious diseases caused by agents not transmitted by vectors, can be appropriately modeled in a guinea pig [7,8,9]. *Rickettsia parkeri*, a tick-borne agent of spotted fever rickettsiosis, is transmitted by the Gulf Coast tick, *Amblyomma maculatum*, in the eastern and primarily Gulf Coast states of the United States. Rickettsial load and immune responses to *R. parkeri* have been investigated in the murine skin in the presence of ticks or tick saliva. However, interpretation of the data was limited in part by the lack of clinically relevant signs in the murine model and assays that directly evaluate infiltration of leukocytes, including T cell subsets in most studies [10,11,12].

Several previous studies of skin lesions utilized a murine model in part due to low costs and easy availability [2,11]. However, the guinea pig may still be a superior model for infectious and other diseases because, compared to other rodents, its immune system more closely resembles that of a human [13]. For our host–tick–*Rickettsia* system, guinea pigs are suitable for tick feeding, and their large size allows for the collection of multiple skin biopsies without needing to sacrifice the animal. In 2019, Saito et al. described a tick transmission model using C3H/HeN mice, with systemic infection confirmed by the presence of rickettsiae in tissues from mice exposed to nymphal *A. maculatum* ticks infected with *R. parkeri* and the development of eschar-like lesions [14]. Still, despite the capacity of strain C3H/HeN to acquire infection via tick transmission, significant differences in the murine and human immune systems as cited above suggest mice are not ideal models for infectious agents, including spotted fever group rickettsiae, supporting the need to further develop the guinea pig as an alternative model [13,15].

Here, we present a novel six-color/eight-parameter flow cytometry assay for monitoring leukocyte infiltration at the site of a skin lesion, specifically an area of inflammation after the bite of a tick with *R. parkeri* during early eschar development on a guinea pig. We believe that this assay enhances the value of the guinea pig model for spotted fever rickettsiosis and other infectious disease research, especially those diseases resulting in dermal lesions.

## 2. Results and Discussion

**Figure 1:**
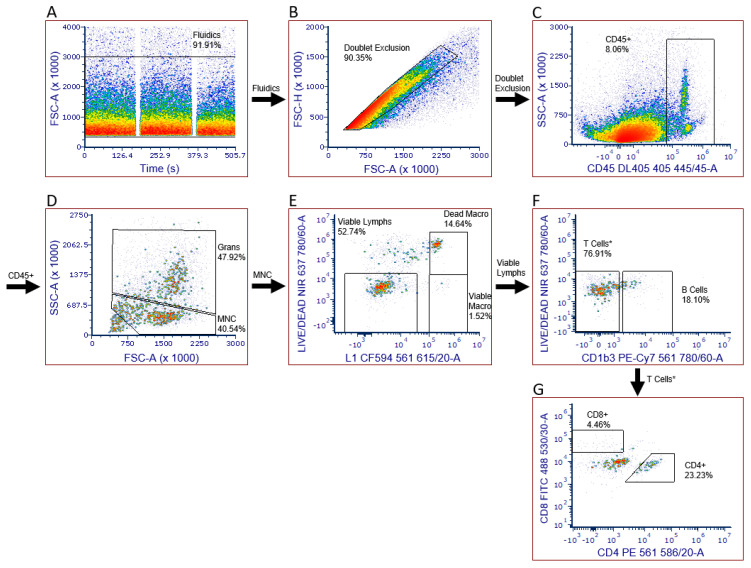
Gating strategy for identifying leukocytes in guinea pig skin. (**A**) Assessed fluidics to ensure that bubbles, microclogs, or other evidence of instability were not present; (**B**) eliminated doublets; (**C**) eliminated skin cells; (**D**) eliminated debris and granulocytes to obtain a population of mononuclear cells (MNC); (**E**) identified viable lymphocytes, viable macrophages, and dead macrophages using a viability stain; (**F**) excluded B cells to obtain a population (T cells *) made up of mainly T cells but likely including a few Kurloff, dendritic, and natural killer cells; (**G**) positive identification of T cell subsets.

We developed a six-color/eight-parameter flow cytometric panel to immunophenotype infiltrating leukocytes at the site of a guinea pig skin lesion; the gating strategy is shown in Figure 1. After initially starting with healthy skin on guinea pigs and then testing the panel on skin from guinea pigs exposed to ticks infected with the spotted fever group rickettsial pathogen, *R. parkeri*, we identified populations of leukocytes, including T cell subsets and macrophages. We developed this assay by optimizing enzyme concentrations for tissue dissociation, titrating antibodies to determine the optimal concentrations, and including appropriate reagents to block both Fc receptor binding of antibodies and non-specific binding of the cyanine fluorophore in PE-Cy7 to monocytes. We subjected all compensation controls except the LIVE/DEAD compensation control to the same experimental conditions as all other samples. The beads used for the LIVE/DEAD compensation control did not tolerate the intracellular staining steps well, so we removed this control from the workflow just prior to fixation. Compensation was not adversely affected, as the emission spectra of this type of dye is not affected by formaldehyde fixation.

To assess the reproducibility of the assay, we performed technical replicates in guinea pig skin that was not exposed to ticks. Briefly, we conducted three consecutive assays, each with three replicates, performed on different days using two different Hartley guinea pigs. Results are provided in Table 1. These samples consisted of a 4-mm guinea pig skin biopsy spiked with 20 µL of guinea pig peripheral blood with erythrocytes lysed. The blood was included so that a reproducible number of leukocytes would be present in each biopsy, which would be expected to have a negligible number of leukocytes in normal tissue.

**Figure 2:**
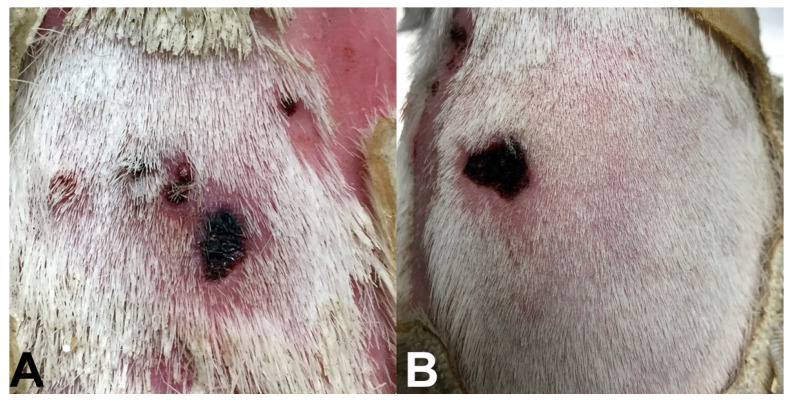
Examples of eschars observed on Hartley guinea pigs exposed to *R. parkeri*-infected *A. maculatum* ticks in unpublished pilot studies within our laboratory. (**A**) Eschar noted on the same day of removal of an *R. parkeri*-infected male tick, 11 days after initial tick placement. (**B**) Eschar noted on a guinea pig 13 days after initial infestation with *R. parkeri*-infected ticks that were removed 12 days after placement.

To determine whether we could use the panel to detect leukocyte infiltration resulting from a bite from a pathogen-infected tick, we used three skin biopsies taken from the sites of tick bites where inflammation was present and two additional control biopsies from areas outside the tick exposure area (chamber) where there was no inflammation evident. We repeated this experiment on a second guinea pig assayed on a different day. Both guinea pigs were tested seven days after tick placement. We selected seven days post-tick placement to capture inflammation of the bite site prior to later stages of eschar development. In pilot studies using Hartley guinea pigs in our laboratory, we found that eschars typically develop 11 to 13 days after initial tick placement (Figure 2). The development of eschars may occur over a period of days to over a week. For example, when cultivated *R. parkeri* (10^5^ rickettsiae) were injected into guinea pigs, an eschar developed in approximately three days [16]. For humans with an infected tick bite, an eschar may take a week or more to develop; for example, in one patient, an eschar developed after the patient removed the tick eight days earlier [17]. Further, in the first confirmed case of *R. parkeri* rickettsiosis in the United States, Paddock describes multiple eschars at various stages of development on physical examination nine days after onset of symptoms [18].

**Figure 3:**
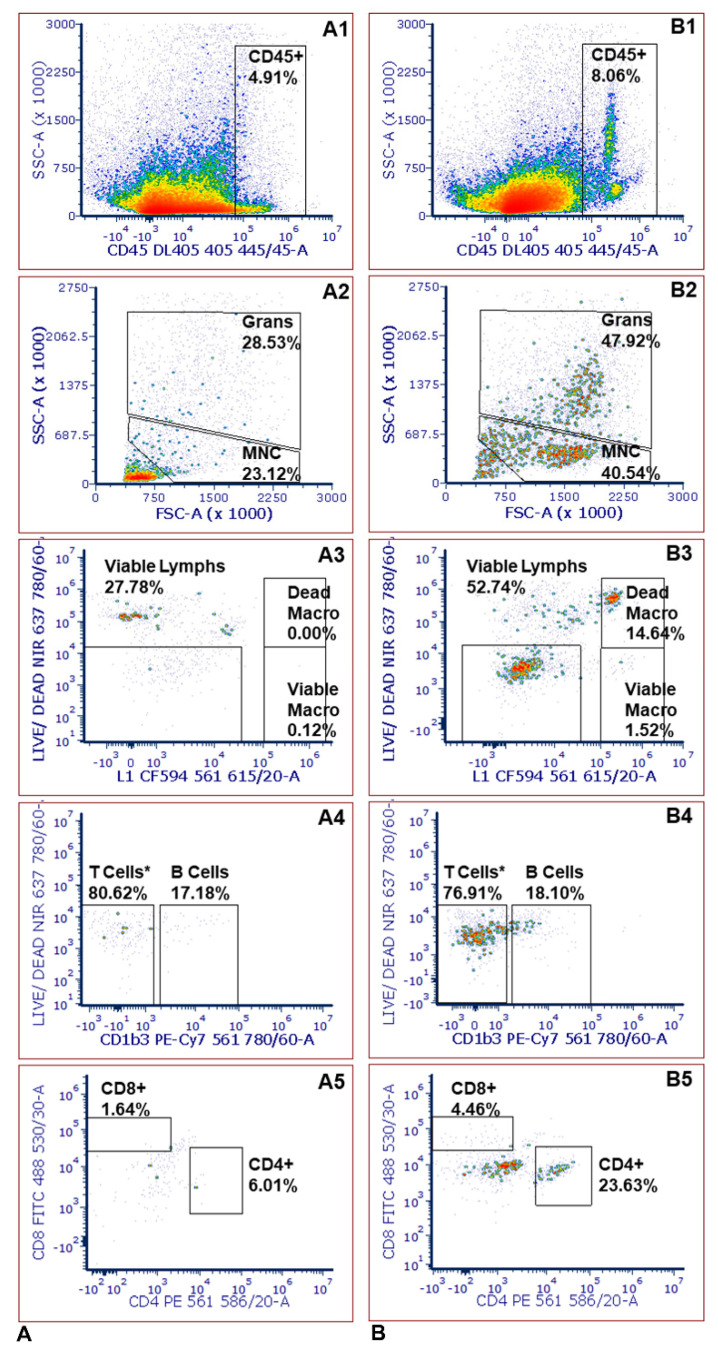
Comparison between skin biopsies taken from the site of a tick bite and control biopsies that did not originate from the site of a tick bite. (**A**) Immunophenotyping from a control biopsy; (**B**) Immunophenotyping from a biopsy taken at the location of a tick bite from the same guinea pig on the same day. MNC stands for mononuclear cells. The T cell * population likely includes some Kurloff, dendritic, and natural killer cells in addition to T cells. Gating was performed as described in Figure 1 in the text, such that: (**A1**,**B1**) gated the cell population after elimination of skin cells; (**A2**,**B2**) correspond to gating to eliminate debris and granulocytes for a population of mononuclear cells (MNC); (**A3**,**B3**) identified viable lymphocytes, viable macrophages, and dead macrophages; (**A4**,**B4**) distinguished B and T cells; (**A5**,**B5**) identifies T cell subsets.

**Figure 4:**
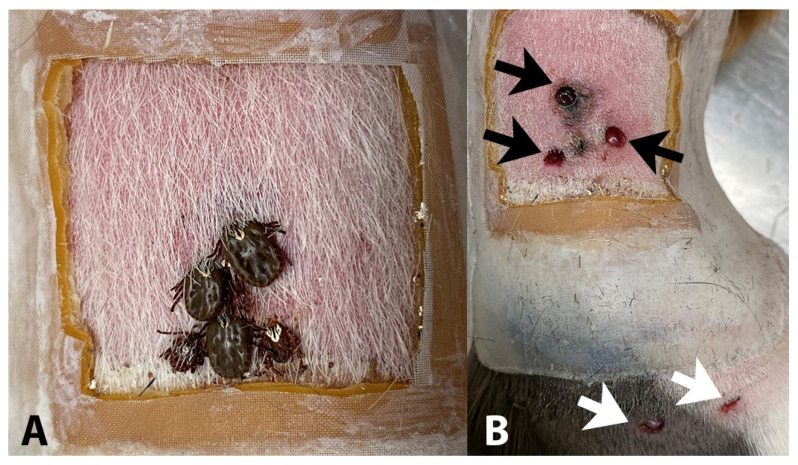
Tick containment chamber and the associated sample collection sites on an inbred guinea pig. (**A**) Four female and one male *R. parkeri*-infected Gulf Coast ticks attached to guinea pig dorsum seven days post-infestation, shown after removal of top layer of containment chamber. (**B**) Skin biopsies taken at tick bite sites (black arrows) and control sites outside of chamber (white arrows) after removal of ticks on the same day as (**A**).

Here, preliminary data show that leukocytes infiltrate the skin following the bite of an *R. parkeri*-infected tick (Figure 3 and Figure 4; Table 2). This infiltration is demonstrated by the increased number of CD45+ cells, granulocytes, and both viable and dead macrophages compared to skin outside of the chamber where ticks did not feed, and inflammation was not grossly evident. Results that led to our final assay are described below.

### 2.1. Tissue Dissociation Optimization

After a discussion with Miltenyi, we determined that Enzyme D was collagenase, Enzyme R was protease, and Enzyme A was DNase I. As the primary enzyme, Enzyme D was optimized first using the concentration of Enzyme R suggested by Miltenyi. Enzyme R was optimized second, using what we found to be the optimal concentration of Enzyme D. We optimized the enzymes stepwise by initially testing concentrations 50% higher than and 50% lower than the recommended concentration. The concentration that yielded the highest number of live cells per milligram of tissue was chosen as the new middle point upon which new concentrations that were 50% higher and 50% lower were calculated and tested. Miltenyi also informed us that Enzyme A is used at a concentration of 100 U/mL. We found that Enzyme A was the limiting reagent in the kit, so to maximize the number of samples that could be digested from one kit of enzymes, the volume of Enzyme A was halved in the cocktail, which resulted in halving the amount of DNase I to 50 units. We replaced the lost volume with an additional 50 U/mL of DNase I to compensate for halving the volume of Enzyme A.

### 2.2. Antibody Titrations

Next, we performed antibody titrations. Peripheral blood was used for titrations and all FMOs in the assay to minimize the number of skin biopsies taken from the guinea pigs. To accurately determine optimal antibody concentrations through titrations necessitated titrating on a cell count approximating what we expected to get from our final assay, where inflammation would be present. We selected an appropriate cell count using a leukocyte infiltration assay performed in rats where one-quarter of all analyzed cells were leukocytes [19]. In our assay development, we consistently achieved cell counts of at least 200,000 cells per 4-mm skin biopsy punch during the tissue dissociation optimization phase. Even with conservative guesses of only 20% of the cells being leukocytes and only 80% of those leukocytes being viable, there should be ~30,000 viable leukocytes per sample. Some cells would also be lost during the washing and decanting steps of sample preparation, so we expected fewer than 30,000 leukocytes per sample. We initially considered titrating using 10 µL of blood, which typically contains ~10,000 white blood cells. This cell number would have been adequate to base our titration on because the cell count in a titration can usually be quartered or quadrupled and still be valid if the staining volume remains constant. However, we obtained low and variable cell counts, so we increased to 20 µL of blood per sample and consistently acquired ~20,000 cells. The blood was lysed and subjected to the same enzymatic dissociation conditions as the tissue samples for antibody titrations.

### 2.3. Leukocyte Infiltration Assays

As expected, the experiments using guinea pigs exposed to *R. parkeri* ticks produced slightly different results because the experimentally infected ticks likely had different bacterial loads when placed on guinea pigs. However, taken together with our technical replicates, data from skin in tick-exposed guinea pigs suggest that our panel both reproducibly and effectively monitors leukocyte infiltration at a skin lesion. The presence of dead macrophages suggests that *R. parkeri* was likely transmitted from the tick to the guinea pig skin, where macrophages would have phagocytosed the rickettsial pathogen, then ultimately undergone cell death. Conversely, several studies show survival of infected macrophages or macrophage-like cells, using in vitro systems with *R. parkeri*-infected cells, such as differentiated THP-1 cells or murine primary cell lines [20,21]. Histopathology of human eschars confirms macrophages in infiltrates after tick transmission [22,23]. Additionally, Banajee et al. [12] found that macrophages, early infiltrates after *R. parkeri* inoculation, phagocytized *R. parkeri*, though this was diminished by the presence of tick saliva in their murine model; however, that study did not include a cell viability stain in the flow cytometry panel. To our knowledge, there are no published studies to date that characterize macrophages after natural tick transmission in humans, guinea pigs, or other models, or that evaluate the viability of macrophages present in similar lesions. Our data warrant further study to determine the role and outcome of macrophages after tick transmission of *R. parkeri*. Notably, some samples taken from the site of a tick bite did not show inflammation. Indeed, tick saliva in the absence of a pathogen has been shown to suppress a local immune response [12]. The second assay (data not shown) had some additional background noise due to blood lysing, so two FMOs had to be set to ~0.50% instead of 0.15% (the B cell FMO was set to 0.50%, and the CD8 FMO was set to 0.48%). However, the placement of those gates matched the clear separation seen in the sample, making those FMOs less critical for determining positivity in that case.

### 2.4. Limitations

One minor limitation of this panel is that we did not include a pan-specific antibody against T cells. This was necessary because the only commercially available pan-specific T cell antibody does not work well in our hands when combined with the intracellular staining and was therefore excluded from the panel. However, our gating strategy nonetheless allowed us to differentiate cytotoxic and helper T cells. Another limitation is that we did not have an antibody to separate the granulocytes from the mononuclear cells. Thus, the current separation strategy for monocytes versus granulocytes is based on scatter properties.

In the leukocyte infiltration assays, the inclusion of additional controls, such as an uninfected tick, would have allowed us to determine how much of the leukocyte response resulted from the invading pathogen and how that differed from the tick bite itself. Additionally, not every tick injected with *R. parkeri* may become infected and subsequently transmit bacteria. In the future, injected ticks will be retained for testing infection status after feeding. Finally, it is likely that biopsies taken at a time-point earlier or later than seven days post-exposure would have shown different infiltration patterns. Maximal infiltration time varies with different pathogens and circumstances (e.g., time of attachment). Thus, these aspects were beyond the scope of this method’s development.

Additionally, in the future, we will address the need for a T cell antibody by modifying the fixation conditions and including an antibody that can distinguish granulocytes from macrophages, so the granulocytes can be more accurately measured. Finally, our goal is to add a novel antibody against spotted fever group *Rickettsia* species that will allow for the detection of *R. parkeri* in macrophages for evaluating successful tick transmission and the initial consequences of rickettsial infection. There are several antibodies we plan to test, including a commercial SFGR-wide antibody and will be exploring other sources, including monoclonal antibodies (e.g., 14-4) used by Anacker et al. [24].

## 3. Materials and Methods

### 3.1. Sample Collection

During initial assay development, skin biopsies and blood samples were collected from approximately 2 to 16-month-old male Hartley guinea pigs (Charles River Laboratories, Wilmington, MA, USA). Three 4-mm biopsy punches were taken from the dorsum of the guinea pig and stored in 250 µL of the MACS Tissue Storage Solution with gentamicin (50 µg/mL) and stored at room temperature (RT) for 1–2 h until tissue dissociation. To test the assay on skin with lesions caused by tick bites, we used 4 to 6-month-old male inbred (Strain 2) guinea pigs. Here, we collected samples seven days after infestation with four female and one male *Amblyomma maculatum* (Gulf Coast tick) that had been experimentally infected by needle injection as engorged nymphs with the spotted fever group *Rickettsia*, *R. parkeri* sensu stricto (Portsmouth isolate; passage 10), as previously described [25]. We purchased engorged nymphs from colonies at Oklahoma State University. The source was selected because of their immediate availability for injection with *R. parkeri* and suitability for the purpose of testing proof of concept of this flow cytometric assay on inflamed guinea pig skin. Pre-screening for pathogens, including spotted fever group *Rickettsia* spp., is a prerequisite for experimental studies utilizing this assay. For samples collected after tick exposure, three biopsy punches were collected within the tick chamber areas at tick bite sites, and two punches were collected outside the chamber on the posterior dorsum where neither ticks nor signs of inflammation were present (Figure 4). Blood was taken from the jugular vein of the guinea pig and stored in EDTA microtainers at RT. All guinea pigs were placed under isoflurane (3%) anesthesia for blood and skin biopsy collection. Guinea pigs were monitored post-recovery, and biopsy samples were collected at least two weeks after the last collection during initial assay development to allow the skin from previous biopsy sites to heal, as per our IACUC protocol. The Mississippi State University Institutional Animal Care and Use Committee (IACUC) approved all procedures following AAALAC guidelines (IACUC protocol numbers 17-166 and 20-210). See Table S1 for a complete list of resources.

### 3.2. Panel Design

Fluorophores were chosen to minimize cross-laser excitation and minimize fluorescence spillover (see Figure S1). Of the five antibodies in the panel, CD45 (DL405), L1 (CF594), and CD1b3 (PE-Cy-7) were labeled in-house; all five antibodies were titrated to determine the optimal concentration (see Figure S2). We included a viability dye to exclude dead cells from the analysis. Guinea pig serum was previously determined to effectively block Fc receptors at a final concentration of 5% in the staining buffer [26], and monocyte blocker was used to prevent non-specific binding of PE-Cy7 to monocytes. We assessed positivity using Fluorescent Minus One (FMO) controls, as seen in Supplementary Figure S3. See Supplementary Table S1 for a complete list of resources.

### 3.3. Tissue Dissociation

Skin biopsies were dissociated with Miltenyi’s Multi Tissue Dissociation Kit 1, which contains Enzyme D (collagenase), Enzyme R (protease), and Enzyme A (DNase). First, we minced the biopsies into approximately fifteen pieces using a scalpel. If a sample had blood on the surface, it was swirled in PBS to rinse off erythrocytes prior to mincing. The tissue samples were transferred to gentleMACS™ C Tubes containing 11.3 µL Enzyme D, 27.6 µL Enzyme R, 7.5 µL Enzyme A, 50 U DNase I, 7.5 µL MgCl2, and complete RPMI (cRPMI) to q.s. to 3 mL. Samples were dissociated at 37 °C using the 37C_Multi_A_01 program (from the default list of programs) on a gentleMACS™ Octo Dissociator with Heaters. We monitored the samples for the entire 41 min of dissociation to ensure that the tissue biopsies remained in the enzyme solution and did not become stuck to the tube wall or rotor. After dissociation, each C Tube was pulsed at 100× *g* to collect the solution at the bottom of the tube. The cell suspension was then passed through a 70 µm strainer. Subsequently, the strainer was washed with 10 mL of warm (37 °C) cRPMI and centrifuged at 350× *g* for 5 min at RT. The cells were washed with 3 mL PBS and then pelleted at 350× *g* for 5 min at RT. See Table S1 for a complete list of resources.

### 3.4. Immunofluorescent Staining

After dissociation, the samples were resuspended in 1 mL of phosphate-buffered saline (PBS) and passed through a 35 µm strainer. Each sample was stained with 1 µL of LIVE/DEAD™ Fixable Near-IR viability dye that had been reconstituted with 100 µL of DMSO. We incubated the cells for 30 min at 4 °C in the dark. Cell suspensions were then centrifuged at 350× *g* for 5 min at RT and washed with 3 mL flow cytometry PBS (FCM-PBS; 1% bovine serum albumin in PBS sterile filtered to 0.1 µm). Guinea pig serum (5%) was added to block Fc receptors, and 5 µL True-Stain Monocyte Blocker™ was added to block non-specific binding of monocytes to PE-Cy7. The cells were then incubated for 30 min at 4 °C in the dark. Next, the cells were surface-stained with antibodies against CD45 (DL405), CD1b3 (PE-Cy7), CD4 (PE), and CD8 (FITC) in a total volume of ~104 µL for 30 min at 4 °C in the dark. Cell samples were washed with 3 mL FCM-PBS and centrifuged at 350× *g* for 5 min at RT, then washed with 3 mL of PBS and centrifuged again at 350× *g* for 5 min at RT. Next, the cells were fixed with 1 mL of 2% formaldehyde in PBS and incubated for 30 min at 4 °C. The cells were then centrifuged at 800× *g* for 5 min at RT. The samples were washed with 2 mL of 1× Intracellular Staining Permeabilization Wash Buffer, incubated for 5 min at RT, and centrifuged at 800× *g* for 5 min at RT. The samples were then washed again with 2 mL of 1× Intracellular Staining Permeabilization Wash Buffer, incubated for 5 min at RT, and centrifuged at 800× *g* for 5 min at RT. Guinea pig serum (5%) was added to block non-specific binding, and the cells were incubated for 30 min at 4 °C. Next, the samples underwent intracellular staining with an anti-L1 (CF594) antibody for 30 min at 4 °C. The cells were then washed with 2 mL of 1× Intracellular Staining Permeabilization Wash Buffer, incubated for 5 min at RT, and centrifuged at 800× *g* for 5 min at RT. Again, the cells were washed with 2 mL of 1× Intracellular Staining Permeabilization Wash Buffer, incubated for 5 min at RT, and centrifuged at 800× *g* for 5 min at RT. Finally, the cells were resuspended in 200 µL FCM-PBS, pelleted at 350× *g* for 5 min at RT, and stored at 4 °C protected from light, to be analyzed within 24 h. See Table S1 for a complete list of resources.

### 3.5. Data Acquisition and Analysis

Immediately before and after each assay, NovoCyte QC particles were used to perform instrument QC. We collected data using a 4-laser, 25-color NovoCyte Quanteon with NovoExpress 1.5.0.2001 acquisition software. Compensation controls and FMOs were included in each assay, and compensation was applied using the software’s automatic compensation tool. We ensured that both the positive and the negative populations for each compensation control contained at least 5000 events. We collected cells from 275 µL of each sample to maximize the number of acquired events. The data were analyzed using FCS Express 7.06.0015. See Supplementary Table S1 for a complete list of resources.

## 4. Conclusions

We developed and optimized a six-color/eight-parameter flow cytometric panel, which allows leukocyte infiltration to be monitored at the site of lesions in guinea pig skin. In the future, our goal is to apply this assay to test hypotheses for leukocyte infiltration in skin where attached ticks are uninfected, infected with pathogenic rickettsiae, or infected with non-pathogenic rickettsiae. Understanding the dermal immune response to ticks in the presence and absence of tick-borne agents will allow us to better understand the mechanisms of immunity in spotted fever rickettsiosis. In addition to its use in the guinea pig-tick-*Rickettsia* system, the assay can be used to study the immune response to other vector-borne diseases, ulcers, and other skin infections that may have previously relied on the use of a murine model.

## Figures and Tables

**Table 1:** Technical replicates were performed using Hartley guinea pig skin biopsies spiked with 20 µL of peripheral blood to simulate leukocyte infiltration. Assays 1 and 3 represent the same guinea pig sampled on different weeks, with technical replicates performed in triplicate for each assay. Gating was performed as described in Figure 1 in the text.

	**Replicate Assay 1**					**Replicate Assay 2**					**Replicate Assay 3**				
	**1**	**2**	**3**	**Mean**	**CV**	**1**	**2**	**3**	**Mean**	**CV**	**1**	**2**	**3**	**Mean**	**CV**
**B%**	13.8	10.7	11.5	12.0	11.1	14.4	14.4	12.9	13.9	5.0	17.1	17.1	18.0	17.4	2.3
**CD4%**	39.5	32.3	36.2	36.0	8.2	38.3	36.8	35.8	37.0	2.8	32.7	32.6	32.2	32.5	0.6
**CD8%**	15.8	16.7	17.2	16.5	3.5	28.8	29.7	29.9	29.4	1.6	19.0	18.4	18.2	18.5	1.9
**CD4/CD8**	2.5	1.9	2.1	2.2	10.9	1.3	1.2	1.2	1.3	4.4	1.7	1.8	1.8	1.8	1.6
**Monocyte%**	39.0	43.5	44.0	42.2	5.3	33.2	32.0	29.5	31.6	4.9	45.9	47.3	47.3	46.8	1.4
**Granulocyte%**	0.81	0.68	0.88	0.79	10.5	0.53	0.72	0.73	0.66	13.9	0.94	0.77	1.08	0.93	13.6

**Table 2:** Comparison between skin biopsies taken from the site of a tick bite and control biopsies that did not originate from the site of a tick bite. For each assay, all biopsies were taken from the same Strain 2 guinea pig, one week after ticks were placed on that guinea pig. Assays were performed on different days with two different Strain 2 guinea pigs, and replicates within assays do not represent technical replicates. The three “tick” biopsies represent biological replicates taken for sites where an individual tick attached, whereas the two “no tick” biopsies represent biological replicates from the skin outside the tick containment area (i.e., negative controls without ticks) for each of the two guinea pigs. Gating was performed as described in Figure 1.

	Assay 1							Assay 2						
	Tick				No Tick			Tick				No Tick		
	1	2	3	Mean	1	2	Mean	1	2	3	Mean	1	2	Mean
**CD45** **(Cell Count)**	10,626	5996	7976	8199	3533	3439	3486	3894	14,000	3847	7247	1901	3061	2481
**B%**	11.3	18.1	10.5	13.3	17.2	9.8	13.5	20.8	15.1	19.3	18.4	16.4	20.3	18.3
**CD4%**	28.8	23.2	34.4	28.8	6.6	4.4	5.5	19.2	28.8	13.2	20.4	3.4	4.2	3.8
**CD8%**	6.6	4.5	8.9	6.7	1.64	2.9	2.3	17.3	10.1	12.2	13.2	19.7	8.4	14.0
**CD4/CD8**	4.4	5.2	3.9	4.5	4.0	1.5	2.8	1.1	2.8	1.1	1.7	0.2	0.5	0.3
**Viable Macrophage%**	0.51	1.52	0.50	0.84	0.12	0.00	0.06	0.24	1.06	1.75	1.02	0.34	0.00	0.17
**Dead Macrophage%**	17.57	14.64	1.09	11.10	0.00	0.11	0.06	1.59	46.00	1.28	16.29	2.07	0.77	1.42
**Granulocyte%**	42.4	47.9	35.4	41.9	28.53	24.1	26.3	26.5	33.5	35.3	31.8	25.5	18.7	22.1

## Data Availability

The data presented in this study are available on request from the corresponding author.

## Supplementary Materials

The following supporting information can be downloaded at: https://www.mdpi.com/article/10.3390/pathogens11020119/s1, Supplementary materials to this article include Figure S1: Panel design; Figure S2: Antibody titrations; Figure S3: Panels for gating and positivity controls. Table S1: Complete list of reagents and resources. In addition to these supplementary materials, a complete step-by-step protocol for this assay will be provided on request.
