# Supplementary material for: Skin in the Game: An Assay to Monitor Leukocyte Infiltration in Dermal Lesions of a Guinea Pig Model for Tick-Borne Rickettsiosis
Source: Pathogens. 2022 Jan 20;11(2):119. doi: 10.3390/pathogens11020119 (PMC8878158; doi:10.3390/pathogens11020119)
Supplement: Supplementary file 1 [file pathogens-11-00119-s001.zip › pathogens-1536197-supplementary.pdf]

Laser	Parameter	Antigen	Fluorochrome
**Violet** 405 nm (mW)	445/45	CD45	DL405
**Blue** 488 nm (mW)	530/30	CD8	FITC
**Yellow-Green** 561 nm (mW)	586/20	CD4	PE
	615/20	L1	CF594
	780/60	CD1b3	PE-Cy7
**Red** 637 nm (mW)	780/60	Viability	Near Infrared

Figure S1. Panel Design. The panel was designed to minimize cross-laser excitation, spillover, and residual donor fluorescence.

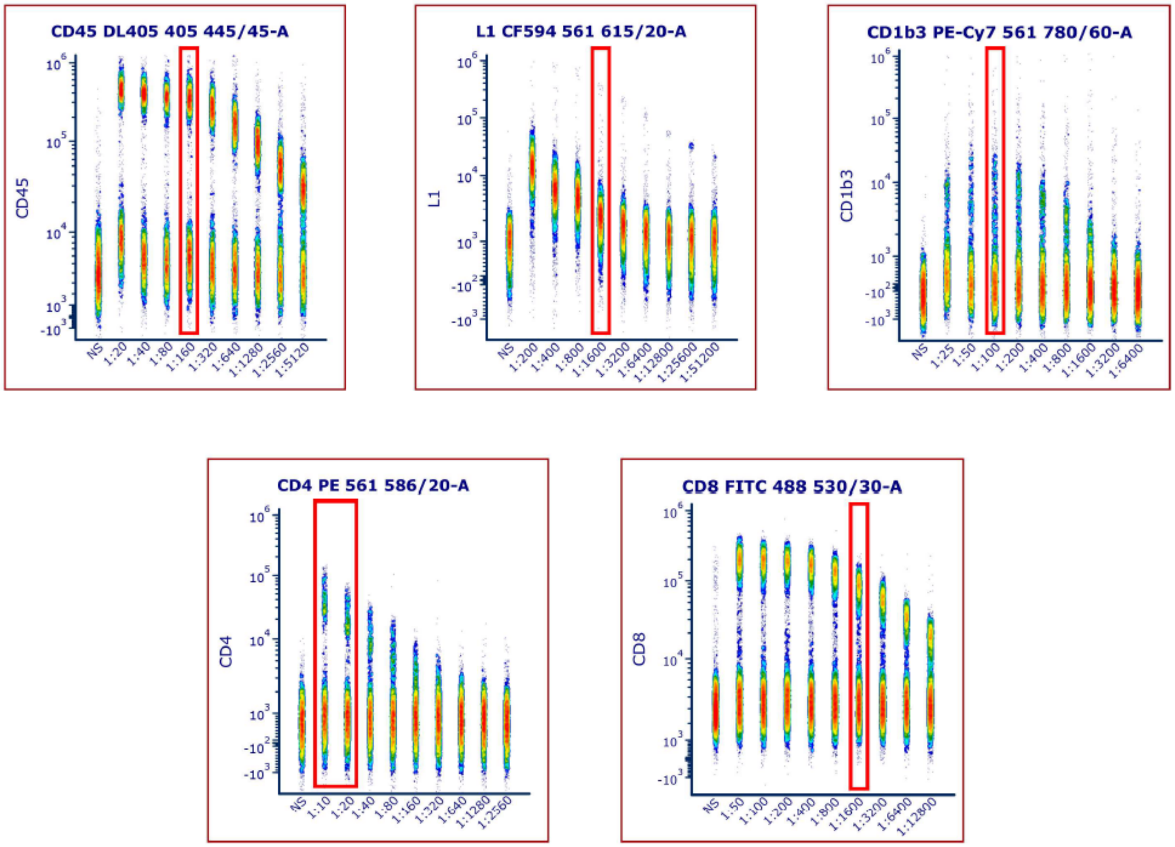

Figure S2. Antibody titrations. Titrations were performed using guinea pig peripheral blood. The protocol was identical to the fully stained tissue samples. The red boxes indicate the selected dilution based on population separation and data spread. On the CD4:PE plot, the chosen dilution was 1:15 due to excessive background noise at the 1:10 dilution. Images show concatenated individual FCS files.

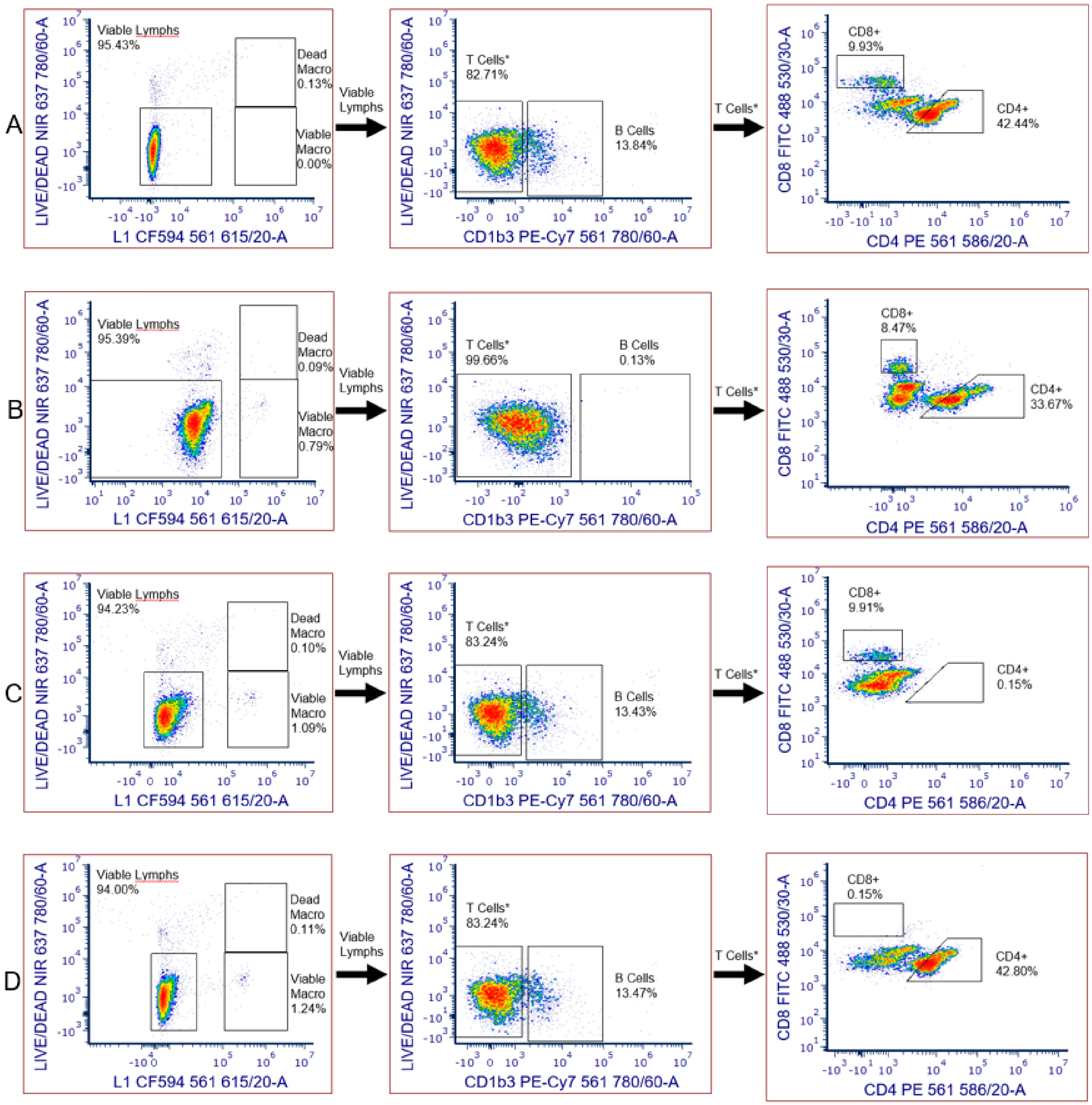

Figure S3. Panels for gating and positivity controls. In these samples, guinea pig peripheral blood was stained with every antibody except for one. All fluorescence minus one (FMO) controls were set to ≤ 0.15%. (A) FMO L1:CF594. (B) FMO CD1b3:PE-Cy7. (C) FMO CD4:PE. (D) FMO CD8:FITC. The T Cells* population likely includes some Kurloff, dendritic, and natural killer cells in addition to T Cells.

Table S1. Complete list of reagents and resources.

REAGENT *or* RESOURCE	VENDOR	CATALOG NUMBER
Tissue Dissociation		
DNase I	Invitrogen	18068015
Fetal Bovine Serum – Hybridoma Qualified, Heat Inactivated	Atlanta Biologicals	S10650H
Gentamicin Solution	Sigma-Aldrich	G1397-10ML
gentleMACS C Tubes	Miltenyi Biotec	130096334
gentleMACS 70 µm SmartStrainers	Miltenyi Biotec	130098462
MACS Tissue Storage Solution	Miltenyi Biotec	130100008
Magnesium Chloride Solution	Sigma-Aldrich	63069-100ML
Multi Tissue Dissociation Kit 1	Miltenyi Biotec	130110201
RPMI 1640 Medium - No Phenol Red	Gibco	11835030
Antibodies and Dyes		
Lightning-Link PE-Cy7 Conjugate Kit	Abcam	ab102903
Lightning-Link Rapid DyLight 405 Conjugate Kit	Abcam	Ab201798
LIVE/DEAD Fixable Near-IR Dead Cell Stain	Invitrogen	L34976
Mix-n-Stain CF594 Antibody Labeling Kit	Biotium	92236
Mouse anti-Guinea Pig CD1b3 (Clone MsGP9)	Bio-Rad	MCA566GA
Mouse anti-Guinea Pig CD4: PE (Clone CT7)	Bio-Rad	MCA749PE
Mouse anti-Guinea Pig CD45 (Clone IH-1)	Bio-Rad	MCA1130
Mouse anti-Guinea Pig CD8: FITC (Clone CT6)	Bio-Rad	MCA752F
Mouse anti-Human (guinea pig-reactive) Macrophages (Clone MAC387)	Bio-Rad	MCA874G
Other Reagents		
Bovine Serum Albumin	Sigma-Aldrich	A3059-100G
Cell Culture Water	Sigma-Aldrich	W3500
DMSO, Anhydrous	Invitrogen	D12345
Guinea Pig Serum	Sigma-Aldrich	G9774-5ML
Intracellular Staining Permeabilization Wash Buffer (10X)	BioLegend	421002
Millipore Purified Water (0.1 µm filtered)	Millipore-Sigma	
NovoCyte QC Particles	Acea Biosciences	8000004
OneComp eBeads Compensation Beads	Invitrogen	01111142
PBS (Ca^{2+} and Mg^{2+} free), pH 7.4	Gibco	10010049
Pierce 16% Formaldehyde (w/v), Methanol-free	Thermo-Fisher	28906
Propidium Iodide Staining Solution	Invitrogen	00699050
Simply Cellular anti-Mouse Beads for Violet Laser	Bangs Laboratories	451
True-Stain Monocyte Blocker	BioLegend	426102
Trypan Blue Stain (0.4%)	Invitrogen	T10282
Viability Dye Compensation Standard (8 µm)	Bangs Laboratories	451
Materials		
Acrocap 0.1 µm Filters	Pall Corporation	4481
Acrodisc 0.2 µm Syringe Filters	Pall Corporation	4652
Countess Cell Counter Chamber Slides	Invitrogen	C10228
Falcon Round-Bottom Polypropylene Tubes (12x75 mm)	Corning Life Sciences	352063
Falcon Round-Bottom Polystyrene Tubes (12x75 mm)	Corning Life Sciences	352032
Falcon Round-Bottom Polystyrene Tubes (12x75 mm) with Cell Strainer Cap	Corning Life Sciences	352235
pH Paper	Whatman	2629990
Sterile Disposable Biopsy Punch	Integra	3334

30 mL Syringe with Luer-Lok Tip	BD Biosciences	309650
Instruments and Software		
Countess II FL Automated Cell Counter	Life Technologies	https://www.thermofisher.com/order/catalog/product/A27974?us&en%22%20\l%20%22/A27974?us&en
EVOS LED Light Cube (RFP)	Invitrogen	AMEP4952
FCS Express (version 7.06.0015)	De Novo Software	https://denovosoftware.com/
gentleMACS Octo Dissociator with Heaters	Miltenyi Biotec	https://www.miltenyibiotec.com/
NovoCyte Quanteon (4025-default configuration)	Agilent	https://www.agilent.com/
NovoExpress (version 1.5.0.2001)	Agilent	https://www.agilent.com/
